# Neonatal transport for convalescing infants: occurrence and barriers reported by providers

**DOI:** 10.1038/s41372-026-02833-4

**Published:** 2026-07-30

**Authors:** Lindsay S. Womack-Martenson, Susan E. Manning, Angela Rohan, Lisa Romero, Shanna Cox, Wanda D. Barfield, Charlan D. Kroelinger

**Affiliations:** 1 Centers for Disease Control and Prevention, Atlanta, GA, USA.; 2 US Public Health Service Commissioned Corps, Rockville, MD, USA.

## INTRODUCTION

Regionalized perinatal systems are designed to provide neonatal risk-appropriate care by aligning infant needs with facility capabilities [1]. One important component of these systems is neonatal back-transport: the transfer of convalescing infants from higher-level neonatal intensive care units (NICUs) to lower-level nurseries for continued care. Back-transport supports availability of NICU beds, resources, and specialized clinical staff for infants with complex needs [1].

Despite these benefits, in 2019, fewer than half of states publicly disseminated statewide guidance related to neonatal back-transport and its reimbursement [2]. Provider-reported practice patterns may highlight facilitators and barriers to implementation. To better understand provider practices within existing hospital systems, we assessed provider-reported experiences with neonatal back-transport, including perceived occurrence and barriers.

## METHODS

We analyzed data from the Spring 2025 DocStyles survey, a cross-sectional, web-based, non-probability panel survey of U.S. healthcare professionals conducted by Porter Novelli (https://styles.porternovelli.com/docstyles/). Eligible participants were currently practicing in the U.S., actively seeing patients, with ≥3 years of clinical experience.

We included pediatricians and nurse practitioners who reported exclusively caring for pediatric patients. We assessed provider-reported frequency of neonatal back-transport and perceived barriers to its implementation, along with provider and practice characteristics (Supplementary Table 1).

All data were deidentified and licensed by the Centers for Disease Control and Prevention (CDC) from Porter Novelli. This activity was reviewed by CDC, deemed not research, and was conducted consistent with applicable federal law and CDC policy.^1^

## RESULTS

Of the 256 providers included, most were pediatricians (97.7%), female (59.8%), and aged 41–60 years (55.1%). Most practiced in suburban (60.9%) and group outpatient (71.5%) settings; 45.3% saw 76–100 patients weekly, and 49.6% practiced medicine >20 years (Supplementary Table 2).

**Fig. 1:**
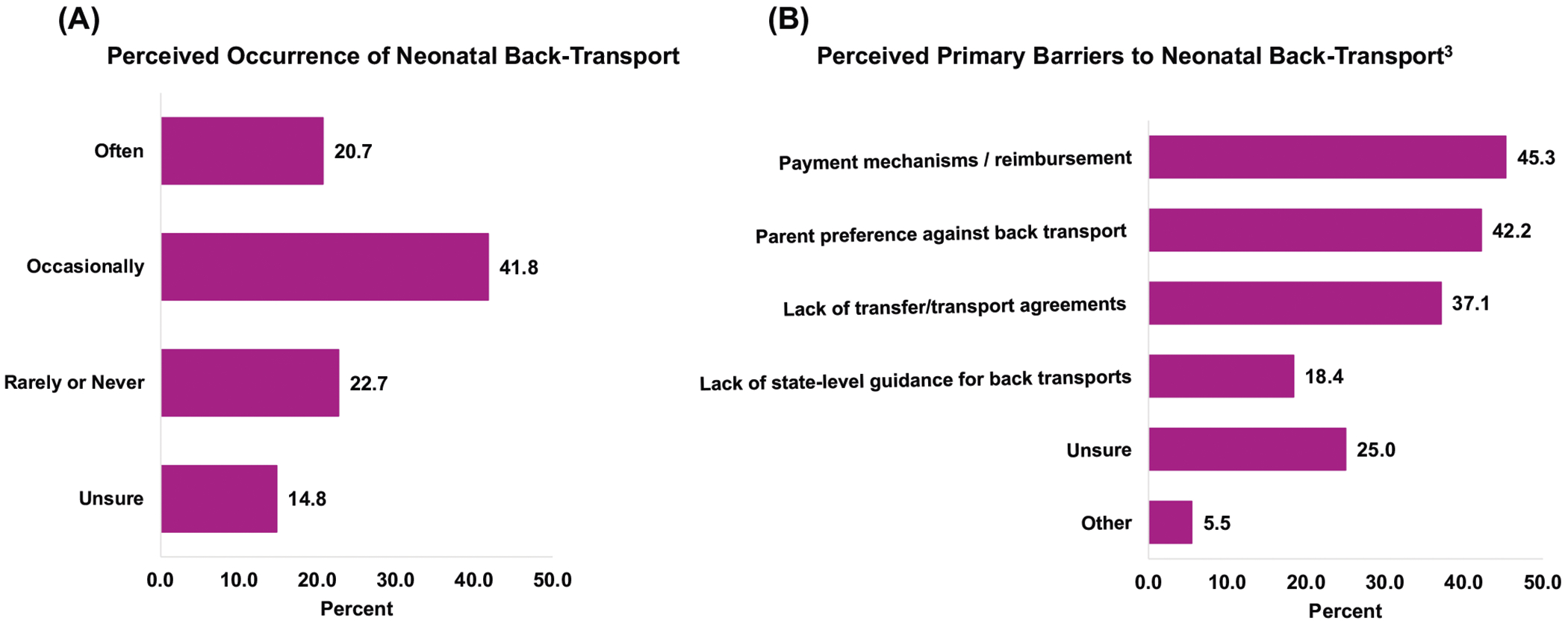
Pediatric provider^1^ perceived occurrence of neonatal back-transport when medically appropriate and reported primary barriers to neonatal back-transport — Spring DocStyles, United States, 2025 (*N* = 256). **A** Perceived occurrence of neonatal back transport. **B** Perceived primary barriers to neonatal back transport. Data are from the Spring 2025 DocStyles survey, a cross-sectional, web-based, non-probability panel survey of U.S. healthcare professionals conducted by Porter Novelli (https://styles.porternovelli.com/docstyles/)^2^. ^1^ Included pediatricians and nurse practitioners who reported exclusively caring for pediatric patients. ^2^ Survey questions are included in Supplementary Table 1. ^3^ Total does not equal 100% because respondents could select multiple responses.

Providers most often reported that neonatal back-transport occurred “occasionally” (41.8%) when medically appropriate, while fewer reported it “often” (20.7%) or “rarely/never” (22.7%) (Fig. 1). Frequently reported barriers included payment mechanisms/reimbursement, parent preference, and lack of transfer agreements (Fig. 1). Patterns were similar when comparing inpatient and outpatient provider responses (Supplementary Table 3).

## DISCUSSION

Our findings suggest that neonatal back-transport occurs infrequently, with only 1 in 5 pediatric providers reporting it occurs “often” when medically appropriate. Most reported that it occurs only “occasionally” or “rarely / never,” despite the benefits for optimizing NICU resources [1]. Perceived barriers included reimbursement, parent preference, and lack of transfer/transport agreements. Notably, one-quarter of providers were unsure about existing barriers, indicating gaps in awareness of back-transport systems.

These results highlight opportunities for addressing barriers to back-transport. Development and consistent implementation of interhospital transfer agreements, standardized protocols, and state-level guidance are critical for ensuring timely access to appropriate levels of neonatal care [1, 2]. Reimbursement was the most frequently reported barrier, which may reflect challenges in aligning financial incentives with regionalized care. Perinatal quality collaboratives (PQCs) and regional networks are well positioned to lead efforts to standardize transfer protocols, address reimbursement barriers, and align policies across hospitals (https://www.cdc.gov/maternal-infant-health/pqc/index.html).

Patient-level barriers, particularly parental preference, were commonly reported. Though many parents ultimately express satisfaction, back-transports are often accompanied by stress, anxiety, and reluctance, particularly when communication is limited or decisions are unexpected [3, 4]. Parents’ concerns often focus on fear of the unknown, loss of trust, and feeling excluded from decision-making [5]. Clear explanations, consistent messaging, and early, family-centered discharge planning improve comfort and acceptance [3]. Psychological comfort with the referring NICU and apprehension about new environments also influence opposition [4]. Early engagement, transparent information, shared planning, and framing back-transport as progress toward discharge can reduce hesitancy and enhance satisfaction [3–5].

Limitations of this analysis include reliance on self-reported perceptions from a convenience sample, which may not represent all pediatric providers. Responses may not reflect actual practices and could be influenced by recall bias or limited knowledge of facility-level practices. Hospital administrators and transport teams were not surveyed, and provider specialty (e.g., neonatology vs. general pediatrics) could not be verified. Only 14.1% of respondents worked primarily in an inpatient practice. However, outpatient and inpatient responses were similar (Supplementary Table 3), suggesting results were not influenced by practice setting. Future research may help further characterize facility-level practices, perspectives from transport teams and other providers involved in neonatal transfer decisions, and family experiences related to neonatal back-transport.

These findings may represent gaps in back-transport awareness, infrastructure, and communication. These efforts could guide protocols, resources, and strategies to strengthen back-transport within neonatal care systems.

## Supplementary Material

Supplementary Material

The online version contains supplementary material available at https://doi.org/10.1038/s41372-026-02833-4.

## Data Availability

Data were derived from the Spring 2025 DocStyles survey, a cross-sectional, web-based, non-probability panel survey of U.S. healthcare professionals conducted by Porter Novelli (https://styles.porternovelli.com/docstyles/).

## Footnotes

1 See e.g., 45 C.F.R. part 46; 21 C.F.R. part 56; 42 U.S.C. §241(d), 5 U.S.C. §552a, 44 U.S.C. §3501 et seq.
