# Supplementary material for: Neonatal transport for convalescing infants: occurrence and barriers reported by providers
Source: J Perinatol. Author manuscript; Available in PMC 2026 Aug 17. (PMC13479253; doi:10.1038/s41372-026-02833-4)
Supplement: Supplementary file 1 [file NIHMS2201333-supplement-Supplementary_Material.docx]

**Supplementary Table 1**. Survey questions to assess pediatric providers’ perceived frequency of neonatal back transport when medically appropriate and reported primary barriers to neonatal back transport — Spring DocStyles, United States, 2025 (N = 256).

| **Survey Question** | **Response Options** |
| --- | --- |
| “In your experience, how often does neonatal back transport occur when medically appropriate?” | Often, Occasionally, Rarely, Never, Unsure |
| What are the primary barriers to neonatal back transport between facilities?” | Select all that apply from: Payment mechanisms/reimbursement, Lack of state policy, Lack of transfer/transport agreements, Parent preference, Other, Unsure |

**Supplementary Table 2**. Prevalence of Provider and Practice Characteristics — Spring DocStyles, United States, 2025 (N = 256).

| **Characteristic** | **Total Sample**  **(N = 256)** |
| --- | --- |
|  | **N (%)** |
| Clinician Type |  |
| Pediatrician | 250 (97.7) |
| Nurse Practitioner | 6 (2.3) |
| Age |  |
| 40 years and younger | 59 (23.0) |
| 41-60 years | 141 (55.1) |
| 61 years and older | 56 (21.9) |
| Sex^1^ |  |
| Female | 153 (59.8) |
| Male | 102 (39.8) |
| Geographical Region of Residence |  |
| Northeast | 92 (35.9) |
| Midwest | 61 (23.8) |
| West | 55 (21.5) |
| South | 48 (18.8) |
| Years in Practice |  |
| 3–10 years | 50 (19.5) |
| 11–20 years | 79 (30.9) |
| 21–30 years | 91 (35.5) |
| 31 years or more | 36 (14.1) |
| Pediatric Patient Volume (Patients/Week) |  |
| 50 or less | 49 (19.1) |
| 51–75 patients | 36 (14.1) |
| 76–100 patients | 116 (45.3) |
| 101 patients or more | 55 (21.5) |
| Approximate household income of majority of patients ($) |  |
| Low income (Less than $25,000) | 14 (5.5) |
| Lower middle ($25,000–$49,999) | 58 (22.7) |
| Middle ($50,000–$99,999) | 83 (32.4) |
| Upper middle ($100,000–$249,999) | 62 (24.2) |
| Upper income ($250,000 or more) | 39 (15.2) |
| Practice Type |  |
| Individual outpatient practice | 36 (14.1) |
| Group outpatient practice or clinic | 183 (71.5) |
| Inpatient practice / hospital | 37 (14.5) |
| Practice Size (Number of Practitioners) |  |
| 1 | 19 (7.4) |
| 2–5 | 71 (27.7) |
| 6–10 | 63 (24.6) |
| 11–15 | 37 (14.5) |
| 16 or more | 67 (26.2) |
| Practice Location |  |
| Urban | 80 (31.3) |
| Suburban | 156 (60.9) |
| Rural | 20 (7.8) |

^1^ Total does not equal 256 due to missing responses.

**Supplementary Table 3**. Pediatric provider perceived frequency of neonatal back transport when medically appropriate and reported primary barriers to neonatal back transport — Spring DocStyles, United States, 2025 (N = 256).

|  | **Inpatient practice / hospital**  **(N = 37)** | **Outpatient Practice or Clinic**  **(N = 219)** | **Overall**  **(N =256)** | **P-value^1^** |
| --- | --- | --- | --- | --- |
|  | **N (%)** | **N (%)** | **N (%)** |  |
| Perceived Frequency of Neonatal Back Transport | | | | |
| Often | 7 (18.9) | 46 (21.0) | 53 (20.7) | 0.53 |
| Occasionally | 18 (48.6) | 89 (40.6) | 107 (1.8) |  |
| Rarely / Never | 9 (24.3) | 49 (22.4) | 58 (22.7) |  |
| Unsure | 3 (8.1) | 35 (16.0) | 38 (14.8) |  |
| Perceived Primary Barriers to Neonatal Back Transport^2^ | | | | |
| Payment mechanisms / reimbursement | 17 (45.9) | 99 (45.2) | 116 (45.3) | 0.93 |
| Parent preference against back transport | 18 (48.6) | 90 (41.1) | 108 (42.2) | 0.39 |
| Lack of transfer/transport agreements | 16 (43.2) | 79 (36.1) | 95 (37.1) | 0.40 |
| Lack of state-level guidance for back transports | 10 (27.0) | 37 (16.9) | 47 (18.4) | 0.14 |
| Unsure | 9 (24.3) | 55 (25.1) | 64 (25.0) | 0.92 |
| Other | 2 (5.4) | 12 (5.5) | 14 (5.5) | 0.99 |

^1^ Two-tailed χ^2^ tests were conducted to compare responses from pediatric providers practicing in inpatient or hospital settings with those in outpatient practices or clinics. Significance was set at *P* < .05.

^2^ Total does not equal 100% because respondents could select multiple responses.
